# *In vitro* digestion and microbial fermentation of dried food residues, a potential “new” component for pet food, and different non-digestible carbohydrate sources

**DOI:** 10.1371/journal.pone.0262536

**Published:** 2022-01-26

**Authors:** Nadine Paßlack, Fenia Galliou, Thrassyvoulos Manios, Katia Lasaridi, Jürgen Zentek

**Affiliations:** 1 Department of Veterinary Medicine, Institute of Animal Nutrition, Freie Universität Berlin, Berlin, Germany; 2 Department of Agriculture, Hellenic Mediterranean University, Heraklion, Crete, Greece; 3 Department of Geography, Harokopio University, Athens, Greece; University of Huddersfield, UNITED KINGDOM

## Abstract

Food residues are often fed to dogs in private households and might also be a potential “new” ingredient for pet food in the future. As food residues might contain not only digestible, but also fermentable substrates, an effect on the intestinal microbiota can be assumed. In the present study, two batches of dried food residues (DFR) collected from hotels in Crete were microbially fermented in an *in vitro* batch culture system with canine fecal inoculum: non-sterile DFR including meat (DFR_m_), sterile DFR including meat (DFR_ms_) and sterile DFR without meat (DFR_wms_). Different non-digestible carbohydrate sources (beet pulp, wheat bran, inulin, carrot pomace, brewer´s spent grains, cellulose and lignocellulose) were included for comparison. Inulin, cellulose and lignocellulose were only used as raw materials, while the other test substrates were incubated as raw and enzymatically pre-digested substrates. After incubation for 24 hours, the raw food residues markedly increased the concentrations of bacterial metabolites in the fermenters, although smaller effects were observed for the DFR_wms_. When the enzymatically pre-digested food residues were incubated, the effects were more pronounced for the DFR_ms_ and DFR_wms_. In general, when compared with the other test substrates, the food residues were microbially fermented to a comparable or partly higher extent. Interestingly, high n-butyrate concentrations were measured in the inocula, both after incubation of the raw and pre-digested food residues. In conclusion, the food residues contained enzymatically digestible and microbially fermentable substrates. If considered as a potential future ingredient for pet food, a standardization of the collection and processing of food residues might be necessary in order to reduce compositional variability and varying effects on the intestinal microbiota.

## Introduction

Given that 1.3 billion tons of food are lost or wasted every year [1], new strategies for food waste reduction are of increasing interest. For instance, the project “Food for Feed (F4F)” (LIFE15 ENV/GR/000257) aims to investigate the potential use of dried food residues (DFR) for animal nutrition. Although legal restrictions currently exist, food residues might be particularly interesting as a potential future ingredient for pet food. In practice, dogs often receive table scraps by their owners [2, 3], making the commercial use of food residues also conceivable.

One major concern of feeding catering waste to animals is its hygienic quality, as several pathogens that could be potentially present in recycled food leftovers may not only be harmful for the animals, but also for human consumers throughout the food chain [4]. A heat treatment of food residues designated for animal nutrition is therefore necessary to ensure the hygienic safety of this material [4].

Another issue of the use of food leftovers for feed is its nutritional composition. Food residues might contain both enzymatically digestible and microbially fermentable substrates. Thus, the dietary inclusion of food residues might also affect the fermentative activity and composition of the intestinal microbiota of animals.

*In vitro* fermentation systems are well established to simulate intestinal conditions and to evaluate the microbial fermentation of certain substrates [5]. As it has been demonstrated that feces are an adequate inoculum [6], these non-invasive models also contribute to the “3R” principle (‘‘reduction, replacement and refinement”) of animal experiments.

In the present study, a batch culture system was used to incubate raw and enzymatically pre-digested food residues with canine fecal inoculum. To compare the effects on the microbial fermentation, different non-digestible carbohydrate sources, varying in their fermentative capacity, were also included. The results of this study should contribute to a better understanding of the effects of food residues on the intestinal microbiota of dogs and might therefore also allow for an evaluation of the suitability of food residues as a potential future ingredient for pet food.

## Material and methods

### Animals and feces collection

Fresh fecal samples were collected from healthy adult dogs kept in the facilities of the Institute of Animal Nutrition, Freie Universität Berlin. All dogs were fed a standard complete dry extruded diet. The dogs were indoor housed with constant light and temperature conditions and had daily access to a clean outdoor area.

### Test substrates

Ten test substrates were microbially fermented in the *in vitro* system: Two different batches of DFR (batch 1: non-sterilized and sterilized DFR including meat (DFR_m_, DFR_ms_); batch 2: sterilized DFR without meat (DFR_wms_)), beet pulp, wheat bran, carrot pomace, brewer´s spent grains, cellulose, lignocellulose and inulin. The composition of the test substrates is presented in Tables 1 and 2. Details on the chemical analyses are provided elsewhere [7, 8].

**Table 1:** Composition (% wet weight) of the food residues used for the present study, and compositional variation (minimum—maximum values) of the food residues collected during the project period^1^.

	Present study		Project period		
	DFR_m_/DFR_ms_	DFR_wms_	Minimum	-	Maximum
Fresh fruits	44.4	46.7	39.7	-	51.3
Cooked meals and snacks	25.4	26.73	19.3	-	32.4
Fresh vegetables and salads	13.9	14.6	9.58	-	17.5
Bread and bakery	5.71	6.00	3.36	-	11.1
Meat and fish	4.90	0.00	3.11	-	8.96
Dairy products (excluding milk) and eggs	0.79	0.83	0.11	-	1.72
Impurities	0.74	0.77	0.32	-	1.42
Sauces, herbs and spices	0.34	0.36	0.00	-	0.90
Desserts	0.22	0.23	0.00	-	0.48
Confectionary and snacks	0.09	0.09	0.00	-	0.35
Processed fruits	0.03	0.03	0.00	-	0.11
Others	3.48	3.66	1.38	-	6.64

^1^ Collection of hotel catering leftovers from autumn 2017—autumn 2018 (n = 4 collection periods); DFR_m_: non-sterile dried food residues with meat (composition already published elsewhere [8]); DFR_ms_: sterile dried food residues with meat; DFR_wms_: sterile dried food residues without meat.

**Table 2:** Analyzed dry matter (DM) and nutrient concentrations of the test substrates used in the present study.

	DFR_m_	DFR_ms_	DFR_wms_	Beet pulp	Wheat bran	Carrot pomace	Brewer´s spent grains	Cellulose	Ligno-cellulose	Inulin
**g/100 g**										
DM	91.2	91.1	86.4	94.1	90.9	94.4	92.4	95.2	90.9	94.1
**g/100 g DM**										
Crude protein	25.9	28.0	31.1	8.62	13.2	10.5	27.9	0.45	0.77	0.14
Crude fat	24.7	23.9	21.5	0.01	1.58	2.33	9.77	0.00	0.37	0.00
Crude fiber	3.46	3.10	4.86	17.3	13.1	22.7	14.4	73.1	65.6	0.00
Crude ash	5.97	6.56	7.98	6.64	1.80	5.10	5.74	0.14	0.41	0.00
Acid detergent fiber	3.84	5.08	7.97	18.3	12.4	28.5	20.9	51.9	73.5	0.00
Neutral detergent fiber	20.3	19.6	20.6	41.2	45.6	44.0	69.8	94.6	94.7	0.00
Soluble dietary fiber	0.81	0.40	1.43	19.4	4.80	21.1	1.76	0.17	1.40	-^1^
Insoluble dietary fiber	10.8	14.0	12.4	47.1	57.4	46.2	53.7	96.6	93.5	-^1^
Total dietary fiber	11.6	14.4	13.8	66.5	62.2	67.3	55.5	96.8	94.9	-^1^
Calcium	0.61	0.58	1.08	1.34	0.04	0.77	0.74	0.02	0.10	0.00
Phosphorus	0.42	0.43	0.47	0.09	0.35	0.17	0.67	0.01	0.01	0.01
Potassium	0.87	1.01	1.36	0.71	0.49	0.89	0.19	0.01	0.04	0.00
Magnesium	0.09	0.09	0.12	0.19	0.12	0.14	0.32	0.01	0.01	0.00
Sodium	0.82	0.94	1.20	0.07	0.01	0.33	0.05	0.03	0.01	0.00

^1^ Below the detection limit (insoluble dietary fiber: 0.380%, total dietary fiber: 0.678%); DFR_m_: non-sterile dried food residues with meat; DFR_ms_: sterile dried food residues with meat; DFR_wms_: sterile dried food residues without meat.

For the compositional analysis of the DFR_m_/DFR_ms_, the ASTM D5231-92 (reapproved 2008) standard [9] was adapted as described by Paßlack et al. [8]. For the production of the DFR_wms_, meat was manually removed from the food residues. The composition of the DFR_wms_ was calculated by determining the relative amount of meat in food residues collected during the analysis period (autumn 2017—autumn 2018) and adjusting the average composition of the collected food residues without meat accordingly. Table 1 also provides data on the compositional variation of the food residues collected during the F4F project period (autumn 2017—autumn 2018) (n = 4 sampling periods).

The food residues were collected from hotel catering in Crete, Greece, ground to a particle size of 10 mm and solar dried in a specific pilot unit in Heraklion, developed in the course of the project “Food for Feed (F4F)” (LIFE15ENV/GR/000257). For the sterilization (DFR_ms_ DFR_wms_), the solar dried samples were treated for 20 minutes at 121°C and 2 bars.

The DFR_m_, DFR_ms_, DFR_wms_, beet pulp, wheat bran, carrot pomace and brewer´s spent grains were added to the *in vitro* system both as raw material and enzymatically pre-digested substrate. Cellulose, lignocellulose and inulin were added as raw material without enzymatic pre-digestion. All the raw substrates were ground at a particle size of 0.5 mm. Fecal suspension without a test substrate was incubated as a blank control.

### Enzymatic pre-digestion of the test substrates

To simulate the microbial fermentation of the substrates in the large intestine, i.e., after digestion by mammalian enzymes, the test substrates were enzymatically pre-digested using a modified method based on the studies of Gauthier et al. [10], Savoie and Gauthier [11] and Minekus et al. [12]. For each test substrate, the enzymatic pre-digestion was performed with 4 replicates. Simulated Gastric Fluid (SGF) and Simulated Intestinal Fluid (SIF) were prepared as described by Minekus et al. [12]. The SIF was stored overnight at 37°C before use.

As a first step, 0.5 g test substrate was mixed with 3 ml SGF. Then, 1 μl CaCl_2_ (0.3 M) was mixed in, and a pH of 3 was adjusted by adding HCl (6 M). Subsequently, 400 μl porcine pepsin (100 mg/ml, dissolved in SGF; activity of porcine pepsin: at least 250 U/mg, according to the manufacturer, Sigma-Aldrich/Merck KGaA, Darmstadt, Germany) were added, mixed, and the solution was filled up to 5 ml with ultrapure water. This solution was mixed and incubated at 37°C for 2 hours in an incubation shaker (Heidolph Inkubator 1000 and Heidolph Unimax 1010, Heidolph Instruments GmbH & CO. KG, Schwabach, Germany).

To stop the pepsin digestion, NaOH (1 M) was added to the solution to adjust a pH of 7. Afterwards, 2 ml of the SIF solution were added, mixed and 1.25 ml porcine bile extract (100 mg/ml, dissolved in SIF) were added. After mixing, 10 μl CaCl_2_ (0.3 M) were added and mixed again. Subsequently, 1.25 ml pancreatin from porcine pancreas (160 mg/ml, dissolved in SIF; pancreatin from porcine pancreas 8 × USP specifications, Sigma-Aldrich/Merck KGaA, Darmstadt, Germany) were mixed to the solution. The pH of the solution was adjusted to 7 using NaOH, and the solution was finally filled up with ultrapure water to 10 ml. After incubation at 37°C for 2 hours in an incubation shaker (Heidolph Inkubator 1000 and Heidolph Unimax 1010, Heidolph Instruments GmbH & CO. KG, Schwabach, Germany), the pancreatin digestion was stopped by incubating the samples on ice for 30 minutes.

In a last step, the samples were washed. For this, dialysis membranes (Spectra/Por^®^ 7 MWCO 1000, 38 mm, Carl Roth, Karlsruhe, Germany) were soaked in water for 15 minutes first. The low ends of these membranes were sealed (Spectra/Por^®^ Universal, 50 mm, Carl Roth, Karlsruhe, Germany), and the enzymatically pre-digested samples were pipetted into the membranes. Afterwards, the top ends of the membranes were also sealed (Spectra/Por^®^ Universal, 50 mm, Carl Roth, Karlsruhe, Germany). The membranes were incubated in 5 l water at 4°C for 24 hours using a magnetic stirrer (IKA RH-KT/C, Sigma-Aldrich/Merck KGaA, Darmstadt, Germany). During the incubation time, the water was changed once. After the incubation, the membranes were opened and the samples were quantitatively transferred into 50 ml tubes. The samples were deep frozen at -80°C and freeze-dried afterwards (Alpha 1–4 LSC, Martin Christ Gefriertrocknungsanlagen GmbH, Osterode am Harz, Germany).

To prove the efficiency of the pre-digestion, the crude protein amount in the inoculum was measured before and after the enzymatic treatment, and the crude protein digestibility (%) was calculated as follows: 100—((protein amount in the inoculum after the pre-digestion (g) / protein amount in the inoculum before the pre-digestion (g)) * 100).

The results of the protein digestibility measurements are presented in Table 3. Due to the neglectable protein amounts present in cellulose, lignocellulose and inulin, the protein digestibility was not calculated for these substrates.

**Table 3:** Calculated protein digestibility of the test substrates^1^ after the enzymatic pre-digestion, but before the microbial fermentation. Means and pooled standard error of the means (SEM).

	Protein digestibility (%)
DFR_m_	73.8
DFR_ms_	76.8
DFR_wms_	69.2
Beet pulp	37.6
Wheat bran	80.8
Carrot pomace	34.2
Brewer´s spent grains	84.4
Pooled SEM	3.77

^1^ Not calculated for cellulose, lignocellulose and inulin, as these substrates contain only small amounts of protein (see Table 2). DFR_m_: non-sterile dried food residues with meat; DFR_ms_: sterile dried food residues with meat; DFR_wms_: sterile dried food residues without meat.

Given the small quantities of substrates used for the pre-digestion trials, and that the pre-digested material was mainly used for a microbial fermentation afterwards, only the protein digestion was calculated as main variable of the pre-digestion, but not the starch or fat digestibility additionally.

### Microbial fermentation

For the microbial fermentation, the protocol of Vierbaum et al. [13] was slightly modified by using 0.5 g of each raw test substrate or the remaining substrate after enzymatic pre-digestion, respectively for the fermentation. The test substrates were weighed in filter bags (Ankom Fiber Filter Bags, F57, ANKOM Technology, Macedon NY, USA).

In a first step, 4 g fresh feces were weighed in 50 ml tubes each. The following steps were performed under anaerobic conditions. The feces were diluted (1:10) with PRAS medium (in g/l aqua bidestillata: 0.5 g KH_2_PO_4_, 0.5 g K_2_HPO_4_, 5.0 g NaHCO_3_, 1.0 g NaCl, 0.1324 g CaCl_2_ x 2 H_2_O, 0.1 g MgSO_4_ x 7 H_2_O, 500 μl Resazurine (0.2%), 5.0 g cysteine hydrochloride; sterilized for 15 minutes at 121°C [14]) and mixed for 2 minutes. After sedimentation for 10 minutes, the supernatant of all tubes was pipetted into one sterile bottle and mixed afterwards (fecal suspension).

In a next step, 90 ml PRAS medium were pipetted into 125 ml afnor bottles (Zscheile & Klinger GmbH, Hamburg, Germany). Afterwards, one welded filter bag with test substrate was placed into a bottle, and 10 ml of the fecal suspension were added. As a blank control, a filter bag without test substrate was placed into a bottle with PRAS medium and fecal suspension. The bottles were sealed and incubated for 24 hours in a waterbath (37°C) and an incubation shaker (Heidolph Inkubator 1000 and Heidolph Unimax 1010, Heidolph Instruments GmbH & CO. KG, Schwabach, Germany).

For each test substrate and blank control, the microbial fermentation was performed in 4 replicates on different days.

### Gas production

For the measurement of the gas volume in the bottles after incubation, a burette (50 ml) was connected with a separation funnel by a tube. The burette was filled with water up to the zero graduation. A canula was connected with the burette by a tube. When the canula was perforating the cover of the incubation bottles, the gas volume in the bottles could be measured by the water displacement from the burette into the separation funnel.

### pH measurement and sample collection

After the measurement of the gas production, the incubation bottles were placed on ice for 30 minutes. The bottles were then opened and the pH was measured in the fecal suspension using a pH meter (Seven Multi, Mettler-Toledo GmbH, Schwerzenbach, Switzerland). One ml aliquots of the fecal suspension were stored at -20°C until further analysis of bacterial metabolites.

### Dry matter loss of the test substrates after incubation

The filter bags were weighed before incubation (tare weight). In addition, the amount of test substrate filled into the filter bag was weighed (t_0_). After the incubation, the welded filter bags, which included the fermented test substrates, were cleaned with distilled water. The filter bags were predried with a tissue and placed into acetone for 5 minutes to remove the remaining fluid. The bags were dried in a compartment dryer at 104°C overnight (Heraeus T5042, Heraeus, Hanau, Germany). After cooling in a desiccator (Duran, DN 300 Novus Duran, Wertheim, Germany), the weight of the welded filter bags was determined. The dry matter loss of the test substrates was calculated as follows:

1. Correction factor for the tare weight of the filter bags after incubation: c = weight (g) of the blank control filter bag after incubation/weight (g) of the blank control filter bag before incubation
2. Weight of the test substrate after incubation (g): t_1_ = weight (g) of the welded filter bag after incubation–(tare weight of the filter bag before incubation (g) * c)
3. Dry matter loss of the test substrate (%) = 100 –(t_1_ (g)/ t_0_ (g) * 100)

### Bacterial metabolites in the fecal suspension after incubation

After thawing of the frozen aliquots, the fecal suspension was centrifuged at 14.000 x g and 20°C for 10 minutes (Thermo Scientific Heraeus Fresco21, Thermo Fisher Scientific, Waltham, MA, USA). Afterwards, 200 μl of the supernatant were mixed with 100 μl hexanoic acid (5 mmol/l, internal standard). The mixture was filled up to 1 ml with oxalic acid (1% w/v), and the concentrations of short-chain fatty acids (SCFA) in the solution were subsequently measured using a gas chromatograph (Agilent Technologies 6890N, auto sampler G2614A, injection tower G2613A, Network GC Systems, Böblingen, Germany) and a polyethylene column (Agilent 19095N-123 HP-INNOWAX, Agilent Technologies, Böblingen, Germany).

For the measurement of D- and L-lactate, 500 μl of the fecal suspension were mixed with 500 μl CuSO_4_ solution (0.5 mmol/l). Subsequently, 100 μl of Carrez I solution (17 g zinc chloride in 100 ml purified water) and 100 μl of Carrez II solution (15 g potassium ferrocyanide (II) in 100 ml purified water) were added. The samples were centrifuged at 14.000 x g and 4°C for 10 minutes (Thermo Scientific Heraeus Fresco21, Thermo Fisher Scientific, Waltham, MA, USA), and the supernatant was filtered through a syringe filter (0.2 μm). The lactate concentrations in the solution were measured using high-performance liquid chromatography (HPLC Agilent 1100, Agilent Technologies, Böblingen, Germany; pre-column Phenomenex C 18, 4.0×2.0 mm, Phenomenex Ltd., Aschaffenburg, Germany; analytical column Phenomenex Chirex 3126 (D)-penicillamine, 150×4.6 mm, Phenomenex Ltd., Achaffenburg, Germany).

For the determination of ammonium, the fecal suspension was centrifuged at 14.800 x g and 20°C for 10 minutes (Thermo Scientific Heraeus Fresco21, Thermo Fisher Scientific, Waltham, MA, USA), and the supernatant was diluted (1:90 and 1:100) with 100 mM 3-(N-morpholino)propanesulfonic acid (pH 6.8). Twenty μl of this mixture were pipetted into the wells of a microtiter plate. One hundred μl phenol nitroprusside and 100 μl alkaline hypochlorite were added into each well afterwards. Resulting from the Berthelot reaction, indophenol was formed, and the extinction was measured every 1.3 minute for 20 minutes at 420 nm (Tecan MPlex Microplate Reader, Tecan Austria GmbH, Grödig, Austria).

### Statistical data analysis

The data were analyzed using SPSS 27 (SPSS Inc., Chicago, Illinois, USA), and are presented in tables as means and the pooled standard error of the means (SEM). For group comparisons, a one-factorial analysis of variance (fixed factor test substrate) and Scheffe´ (variance equality) or Tamhane 2 (variance inequality) post hoc tests were considered. Different letters in the same row indicate significant group differences (*P* < 0.05). For the comparison of the raw and enzymatically pre-digested substrates, normality of the data was tested (Kolmogorov-Smirnov and Shapiro Wilk tests), and groups were compared using the t test (parametric data) or Mann-Whitney U-test (nonparametric data).

## Results

### Microbial fermentation of the raw test substrates

The gas production was lowest, when no test substrate was incubated in the canine fecal suspension (blank control), and highest, when the DFR_ms_ were microbially fermented (Table 4). A low gas production was also observed, when cellulose and lignocellulose were incubated, while especially the incubation of DFR, beet pulp, wheat bran and carrot pomace resulted in a high gas production (*P* < 0.05, when these test substrates were compared with the blank control and cellulose incubation).

**Table 4:** Gas production, pH, and microbial metabolites in canine fecal suspension after incubation with different raw test substrates, as well as dry matter (DM) loss of the test substrates after incubation. Means and pooled standard error of means (SEM).

	Blank control^1^	DFR_m_	DFR_ms_	DFR_wms_	Beet pulp	Wheat bran	Carrot pomace	Brewer´s spent grains	Cellulose	Ligno-cellulose	Inulin	SEM
Gas (ml)	6.56^a^	38.3^be^	47.1^efg^	33.0^bdg^	30.4^bdg^	28.7^bc^	28.8^bc^	16.6^acd^	9.64^a^	11.2^ac^	21.3^abcd^	1.78
pH	7.41	6.59	6.61	6.67	6.60	6.82	6.73	7.00	7.29	7.32	6.81	0.06
DM loss of the test substrate (%)	-	58.6	55.6	56.9	44.1	33.8	33.7	15.6	4.99	10.2	67.1	3.11
**μmol/ml**												
Ammonium	10.5^abc^	22.7^b^	20.6^bc^	14.3^a^	12.5^a^	18.6^abc^	14.4^ac^	16.0^abc^	13.0^abc^	12.8^abc^	10.5^a^	0.66
L-lactate	0.03^a^	2.02^b^	1.90^bd^	2.33^be^	0.73^ac^	0.84^acd^	0.96^ce^	0.23^a^	0.03^a^	0.03^a^	0.76^acd^	0.11
D-lactate	0.03^a^	1.43^e^	1.63^e^	1.76^ec^	0.53^ac^	0.44^ad^	0.65^bcd^	0.35^ac^	0.02^a^	0.04^a^	1.10^abe^	0.09
Acetate	1.32^a^	10.2^abc^	9.76^bc^	6.86^bc^	9.05^b^	7.79^abc^	6.51^bc^	4.32^abc^	1.49^ac^	1.86^ac^	3.21^abc^	0.49
Propionate	0.16	1.98	1.90	1.52	1.91	1.44	1.46	0.54	0.20	0.26	1.03	0.11
i-butyrate	0.05	0.27	0.37	0.12	0.13	0.16	0.19	0.08	0.16	0.04	0.12	0.03
n-butyrate	0.12^a^	4.50^b^	3.77^bc^	2.27^abc^	1.34^a^	1.81^ac^	1.10^a^	0.39^a^	0.15^a^	0.13^a^	0.41^a^	0.21
i-valerate	0.02	0.09	0.05	0.05	0.02	0.07	0.01	0.08	0.12	0.06	0.04	0.01
n-valerate	0.00	0.07	0.08	0.11	0.02	0.29	0.02	0.01	0.00	0.00	0.01	0.02
Total SCFA	1.67^a^	17.1^b^	15.9^b^	10.9^bcde^	12.5^bd^	11.6^abcde^	9.30^bde^	5.43^ae^	2.12^ac^	2.37^ac^	4.82^ad^	0.78
**Mol %**												
Acetate	74.5	58.9	60.6	62.8	72.4	67.1	69.4	78.5	70.5	76.3	64.0	1.25
Propionate	10.9^a^	11.5^a^	11.8^a^	14.1^ab^	15.2^ab^	12.6^a^	15.9^ab^	10.2^a^	9.87^a^	11.3^a^	21.3^b^	0.55
i-butyrate	4.41	1.95	2.75	1.33	1.13	1.79	2.37	1.87	6.49	2.34	4.33	0.55
n-butyrate	8.66^a^	26.7^b^	24.0^bd^	20.4^bc^	11.0^ac^	15.3^acd^	12.0^ac^	7.52^a^	7.83^a^	6.08^a^	8.99^a^	1.01
i-valerate	1.18	0.58	0.32	0.52	0.14	0.74	0.05	1.72	5.17	3.67	1.09	0.33
n-valerate	0.32	0.45	0.46	0.90	0.18	2.55	0.26	0.24	0.17	0.27	0.24	0.21

^1^Incubation without test substrate; DFR_m_: non-sterile dried food residues with meat; DFR_ms_: sterile dried food residues with meat; DFR_wms_: sterile dried food residues without meat; SCFA: short-chain fatty acids; Different letters in the same row indicate significant group differences (*P* < 0.05).

The microbial fermentation of the raw test substrates did not affect the pH in the inocula.

The highest ammonium concentrations were measured in the inocula, when the DFR_m_ and DFR_ms_ were incubated, with group differences compared to inulin, beet pulp and DFR_wms_.

The incubation of the DFR_m_, DFR_ms_ and DFR_wms_ also resulted in the highest L-lactate concentrations in the inoculum, and differed compared to the blank control, cellulose, lignocellulose, brewer´s spent grains and beet pulp. A comparable effect was observed for the D-lactate concentrations in the inoculum, with highest concentrations after incubation of the DFR_m_, DFR_ms_ and DFR_wms_, and lower concentrations after the blank control, cellulose, lignocellulose and wheat bran treatment. The D-lactate concentrations were also higher, when the DFR_m_ and DFR_ms_ were incubated when compared to the brewer´s spent grains, carrot pomace and beet pulp fermentation.

The acetate concentrations were low in the blank control (mean 1.32 μmol/ml) and differed after the microbial fermentation of carrot pomace, beet pulp, DFR_ms_ and DFR_wms_ (means 6.51–9.76 μmol/ml). The concentrations of propionate, i-butyrate, i-valerate and n-valerate in the inocula were not different among the groups. Higher n-butyrate concentrations were observed after incubation of the DFR_m_ and DFR_ms_ when compared to the blank control, cellulose, lignocellulose, brewer´s spent grains, inulin, carrot pomace and beet pulp treatment. The concentrations of total SCFA were low in the blank control (mean 1.67 μmol/ml), but higher, when the DRF_m_, DFR_ms_, DFR_wms_, beet pulp and carrot pomace were microbially fermented (means 9.30–17.1 μmol/ml).

When the relative amount of the single SCFA (% of total SCFA) in the inocula was calculated, no group differences could be detected for acetate, i-butyrate, i-valerate and n-valerate. Higher relative amounts of propionate were measured after the microbial fermentation of inulin (mean 21.3 mol %) when compared to the blank control, cellulose, lignocellulose, brewer´s spent grains, wheat bran, DFR_m_ and DFR_ms_ treatment (means 9.87–12.6 mol %). The microbial fermentation of the DFR_m_, DFR_ms_ and DFR_wms_ resulted in the highest relative amounts of n-butyrate (means 20.4–26.7 mol %), while lower amounts of n-butyrate were measured after the blank control, cellulose, lignocellulose, inulin and brewer´s spent grains treatment (means 6.08–8.99 mol %). In addition, the relative amounts of n-butyrate were higher after the microbial fermentation of the DFR_m_ and DFR_ms_ when compared to the inoculation of carrot pomace and beet pulp.

### Microbial fermentation of the enzymatically pre-digested test substrates

The microbial fermentation of the enzymatically pre-digested test substrates resulted in a higher gas and ammonium production compared to the blank control (Table 5). The pH in the inoculum was comparable among all groups.

**Table 5:** Gas production, pH, and microbial metabolites in canine fecal suspension after incubation with different enzymatically pre-digested test substrates, as well as dry matter (DM) loss of the test substrates after incubation. Means and pooled standard error of means (SEM).

	Blank control^1^	DFR_m_	DFR_ms_	DFR_wms_	Beet pulp	Wheat bran	Carrot pomace	Brewer´s spent grains	SEM
Gas (ml)	6.56^a^	36.8^b^	39.1^b^	38.8^b^	38.6^b^	42.0^b^	41.4^b^	37.0^b^	2.11
pH	7.41	6.85	6.73	6.79	6.69	6.50	6.66	6.86	0.06
DM loss (%) of the test substrate	-	75.0	65.8	73.5	61.0	90.2	54.7	55.8	2.38
**μmol/ml**									
Ammonium	10.5^a^	22.6^b^	24.2^b^	28.6^b^	23.5^b^	23.5^b^	24.8^b^	24.6^b^	1.03
L-lactate	0.03^b^	0.09^b^	0.17^b^	0.12^b^	0.23^b^	2.59^a^	0.19^b^	0.09^b^	0.13
D-lactate	0.03	0.32	0.34	0.38	0.32	0.88	0.42	0.24	0.05
Acetate	1.32^a^	9.69^ab^	9.04^ab^	12.4^b^	12.3^b^	10.4^b^	10.9^ab^	8.84^ab^	0.70
Propionate	0.16^a^	0.80^ab^	0.96^b^	1.31^ab^	1.95^b^	1.78^ab^	1.18^b^	0.84^ab^	0.11
i-butyrate	0.05	0.22	0.24	0.24	0.15	0.18	0.21	0.14	0.04
n-butyrate	0.12^a^	2.23^ab^	2.63^b^	2.61^b^	2.63^ab^	4.01^b^	2.13^ab^	2.06^ab^	0.21
i-valerate	0.02	0.10	0.05	0.07	0.05	0.08	0.08	0.07	0.01
n-valerate	0.00	0.01	0.02	0.01	0.01	0.05	0.01	0.01	0.00
Total SCFA	1.67^a^	13.0^ab^	12.9^ab^	16.6^b^	17.1^b^	16.5^b^	14.5^ab^	12.0^ab^	0.96
**Mol %**									
Acetate	74.5	74.5	68.8	74.6	72.4	63.0	74.6	72.8	1.12
Propionate	10.9	6.08	7.66	7.67	11.7	10.4	8.37	7.32	0.45
i-butyrate	4.41	2.06	2.46	1.50	0.97	1.21	1.87	1.91	0.44
n-butyrate	8.66^a^	16.4^ab^	20.5^bc^	15.7^ab^	14.6^ac^	24.5^b^	14.4^ac^	17.2^ab^	0.88
i-valerate	1.18	0.84	0.40	0.45	0.32	0.53	0.69	0.73	0.10
n-valerate	0.32	0.07	0.14	0.08	0.10	0.33	0.11	0.05	0.04

^1^Incubation without test substrate; same blank control as for the raw test substrates (Table 4).

DFR_m_: non-sterile dried food residues with meat; DFR_ms_: sterile dried food residues with meat; DFR_wms_: sterile dried food residues without meat; SCFA: short-chain fatty acids; Different letters in the same row indicate significant group differences (*P* < 0.05).

The concentrations of L-lactate were higher after the microbial fermentation of enzymatically pre-digested wheat bran when compared to all other test substrates and the blank control, whereas the D-lactate concentrations in the inocula did not differ among the groups.

The acetate and total SCFA concentrations in the blank control were lower when compared to the concentrations after the microbial fermentation of the pre-digested wheat bran, beet pulp and DFR_wms_. The propionate concentrations in the inocula were low in general, but higher after the microbial fermentation of enzymatically pre-digested DFR_ms_, carrot pomace and beet pulp when compared to the blank control. Lowest concentrations of n-butyrate were measured in the blank control (mean 0.12 μmol/ml), whereas higher amounts were measured, when pre-digested DFR_ms_, DFR_wms_ and wheat bran were microbially fermented (means 2.61–4.01 μmol/ml). The concentrations of i-butyrate, i-valerate and n-valerate in the inocula did not differ among the groups. When the mol % of the single SCFA was calculated, group differences were only observed for n-butyrate. Highest relative amounts of n-butyrate were measured after the microbial fermentation of enzymatically pre-digested wheat bran (mean 24.5 mol %; group difference compared to the blank control and pre-digested carrot pomace and beet pulp). In addition, the microbial fermentation of enzymatically pre-digested DFR_ms_ also resulted in high relative amounts of n-butyrate (mean 20.5 mol %), which was higher compared to the blank control (mean 8.66 mol %).

### Comparison between the microbial fermentation of the raw and enzymatically pre-digested test substrates

When the microbial fermentation of the raw and pre-digested test substrates was compared, variations in the gas production, DM loss and concentrations of microbial metabolites in the inocula could be observed (Table 6).

**Table 6:** Comparison (*P* values) between the raw and enzymatically pre-digested test substrates (↑ increase or ↓ decrease when compared to the microbial fermentation of the raw test substrate; → no difference between the microbial fermentation of the raw and enzymatically pre-digested test substrate), for means see Tables 4 and 5.

	Raw versus pre-digested test substrate (*P* value)						
	DFR_m_	DFR_ms_	DFR_wms_	Beet pulp	Wheat bran	Carrot pomace	Brewer´s spent grains
Gas (ml)	↓ (0.803)	↓ (0.230)	↑ (0.133)	↑ (0.137)	↑ (0.113)	↑ **(0.006)**	↑ **(0.002)**
pH	↑ (0.184)	↑ (0.587)	↑ (0.526)	↑ (0.703)	↓ (0.090)	↓ (0.728)	↓ (0.542)
Dry matter loss (%)	↑ **(0.011)**	↑ **(0.003)**	↑ **(< 0.001)**	↑ **(0.007)**	↑ **(< 0.001)**	↑ **(0.038)**	↑ **(< 0.001)**
**μmol/ml**							
Ammonium	↓ (0.602)	↑ (0.198)	↑ **(0.001)**	↑ **(0.007)**	↑ (0.175)	↑ **(0.009)**	↑ **(0.016)**
L-lactate	↓ **(< 0.001)**	↓ **(< 0.001)**	↓ **(< 0.001)**	↓ **(0.016)**	↑ **(0.009)**	↓ **(< 0.001)**	↓ **(0.004)**
D-lactate	↓ **(< 0.001)**	↓ **(0.009)**	↓ **(< 0.001)**	↓ (0.090)	↑ (0.174)	↓ (0.067)	↓ (0.149)
Acetate	↓ (0.836)	↓ (0.711)	↑ **(0.003)**	↑ (0.086)	↑ (0.110)	↑ (0.051)	↑ (0.064)
Propionate	↓ **(0.009)**	↓ **(0.017)**	↓ (0.478)	↑ (0.936)	↑ (0.347)	↓ (0.218)	↑ (0.065)
i-butyrate	↓ (0.251)	↓ (0.251)	↑ (0.917)	↑ (0.754)	↑ (0.602)	↑ (0.754)	↑ (0.385)
n-butyrate	↓ **(0.009)**	↓ **(0.035)**	↑ (0.520)	↑ (0.251)	↑ (0.004)	↑ (0.068)	↑ **(0.011)**
i-valerate	↑ (0.465)	→ (0.997)	↑ (0.146)	↑ (0.220)	↑ (0.763)	↑ **(0.007)**	↓ (0.502)
n-valerate	↓ **(0.008)**	↓ (0.113)	↓ (0.071)	↓ (0.738)	↓ (0.602)	↓ (0.447)	→ (0.290)
Total SCFA	↓ (0.233)	↓ (0.218)	↑ **(0.012)**	↑ (0.095)	↑ **(0.047)**	↑ (0.056)	**↑ (0.041)**
**Mol %**							
Acetate	↑ (0.076)	↑ (0.076)	**↑ (0.002)**	→ (0.989)	↓ (0.204)	↑ (0.133)	↓ (0.117)
Propionate	**↓ (0.016)**	**↓ (0.005)**	**↓ (0.002)**	↓ (0.175)	↓ (0.177)	**↓ (0.016)**	**↓ (0.009)**
i-butyrate	↑ (0.917)	↓ (0.347)	↑ (0.602)	↓ (0.602)	↓ (0.917)	↓ (0.754)	↑ (0.347)
n-butyrate	**↓ (0.006)**	↓ (0.124)	↓ (0.094)	↑ (0.238)	**↑ (0.009)**	↑ (0.227)	**↑ (0.009)**
i-valerate	↑ (0.465)	↑ (0.682)	↓ (0.645)	↑ (0.220)	↓ (0.521)	**↑ (0.024)**	↓ (0.117)
n-valerate	**↓ (0.008)**	↓ (0.245)	**↓ (0.023)**	↓ (0.911)	↓ (0.602)	↓ (0.270)	↓ (0.126)

DFR_m_: non-sterile dried food residues with meat; DFR_ms_: sterile dried food residues with meat; DFR_wms_: sterile dried food residues without meat; SCFA: short-chain fatty acids.

For all test substrates, the DM loss was higher after the microbial fermentation of the pre-digested substrates than of the raw test substrates.

The pre-digestion of the DFR_m_ and DFR_ms_ resulted in lower L- and D-lactate, propionate and n-butyrate concentrations as well as in lower relative amounts (mol %) of propionate in the inoculum compared to the microbial fermentation of the raw DFR_m_ and DFR_ms_. In addition, lower total amounts (μmol/ml) of n-valerate and lower relative amounts (mol %) of n-butyrate and n-valerate could be measured in the inoculum after the microbial fermentation of the pre-digested DFR_m_ compared to the microbial fermentation of the raw DFR_m_.

When the enzymatically pre-digested DFR_wms_, beet pulp, carrot pomace and brewer´s spent grains were microbially fermented, higher concentrations of ammonium and lower concentrations of L-lactate were measured than after the microbial fermentation of the raw test substrates. Additionally, the pre-digestion of the DFR_wms_ resulted in lower D-lactate, propionate (mol. %) and n-valerate (mol %) as well as in higher acetate (μmol/ml and mol %) and total SCFA concentrations than after the microbial fermentation of the raw DFR_wms_.

The enzymatic pre-digestion of wheat bran increased the concentrations of L-lactate, total SCFA and the relative amount of n-butyrate in the inoculum. Similar effects were observed for the microbial fermentation of pre-digested brewer´s spent grains, with additionally higher total amounts (μmol/ml) of n-butyrate and lower relative amounts (mol %) of propionate as well as a higher gas production in the inoculum.

The microbial fermentation of pre-digested carrot pomace also resulted in a higher gas production, but additionally in higher total and relative amounts of i-valerate and lower relative amounts of propionate in the inoculum when compared to the microbial fermentation of raw carrot pomace.

## Discussion

Depending on the pattern of bacterial metabolites produced, the microbial fermentation of undigested nutrients can be beneficial, but also detrimental for gut health. While undigested protein entering the large intestine can favor pathogenic bacteria and harmful metabolites of microbial protein fermentation [15], the bacterial fermentation of non-digestible carbohydrates is considered beneficial due to an increased microbial production of SCFA [16] and balancing effects on the intestinal microbiota [17].

In the present study, different non-digestible carbohydrate sources were microbially fermented, using an *in vitro* batch culture system and canine fecal inoculum. On the one hand, the test substrates included dietary ingredients that are highly to moderately fermentable: *inulin*, a prebiotic oligo- or polysaccharide [18, 19], *beet pulp*, containing pectins, cellulose and hemicellulose [20], *carrot pomace* with insoluble and soluble fibers, particularly pectic polysaccharides, hemicellulose and cellulose [21], *wheat bran*, mainly consisting of cell wall polysaccharides like (glucurono)arabino xylans, cellulose and (1→3, 1→4)-beta-glucans, but also of protein and lignin [22], and *brewer´s spent grains*, a by-product of the brewing industry and characterized by high contents of cellulose, non-cellulosic polysaccharides and lignin [23], as well as protein and lipids [24]. On the other hand, substrates that are not or less fermentable were also included: *cellulose*, an insoluble fiber [25], and *lignocellulose*, which mainly comprises cellulose, hemicelluloses and lignin [26]. Different studies have evaluated the microbial fermentation of these test substrates in dogs, both *in vitro* and *in vivo* (e.g. [6, 13, 27–33]). However, the focus of the present study was to evaluate the fermentative capacity of food residues and to compare the effects with the microbial fermentation of the other test substrates. Moreover, as these reference substrates are non-digestible carbohydrate sources, a pre-digestion might not be necessary for their use in an *in vitro* system to simulate the microbial fermentation in the large intestine. In contrast, it was assumed that DFR might not only contain microbially usable substances, but also enzymatically digestible nutrients. Thus, we compared the microbial fermentation of raw and pre-digested substrates in our study to gain more insights into the nutrient profile of DFR as a potential dietary ingredient.

As a main finding of the present study, the raw DFR_m_, DFR_ms_ and DFR_wms_ were highly fermentable, as demonstrated by the highest concentrations of ammonium, lactate, acetate, n-butyrate and total SCFA in the inoculum. Group differences were detected compared to the blank control, but also to other test substrates.

The ammonium concentrations in the inoculum were higher after the microbial fermentation of the raw DFR_m_ compared to the raw inulin, carrot pomace, beet pulp and DFR_wms_. Ammonia is produced by bacterial protein degradation [34] and has been demonstrated to reveal toxic effects in the organism [35]. In healthy individuals, ammonia is detoxified to urea in the liver and excreted by the kidneys afterwards [36].

The higher concentrations of ammonium after inoculation of the raw DFR_m_ might likely result from a higher amount of highly fermentable protein in the raw DFR_m_ compared to the other test substrates. In addition, although the crude protein concentration of the DFR_wms_ was higher than of the DFR_m_, the microbial fermentation of the DFR_wms_ was associated with lower ammonium concentrations in the inoculum. It can therefore be assumed that especially meat protein in the raw DFR_m_ might have contributed to a higher microbial ammonium production. However, as meat protein is highly digestible [37], an inclusion of DFR_m_ in a diet for dogs might not necessarily result in an increased concentration of ammonium in their large intestine. Instead, it can be assumed that meat protein from DFR could be enzymatically digested in the canine small intestine. This assumption is supported by the results of the pre-digestion trials, demonstrating a relatively high crude protein digestibility of the DFR_m_. In addition, the microbial fermentation of the pre-digested DFR_m_ revealed a comparable ammonium production as for the other test substrates, stressing the hypothesis that the raw, but not the pre-digested DFR_m_ contained notable amounts of highly fermentable protein.

The lactate and SCFA concentrations in the inocula were also higher after the fermentation of the raw DFR_m_, DFR_ms_ and, although less pronounced, of the raw DFR_wms_ when compared to most other test substrates. These metabolites result from the bacterial fermentation of non-digestible carbohydrates [38], indicating an intensive microbial degradation of these ingredients of the food residues.

When the enzymatically pre-digested test substrates were microbially fermented, group differences were especially observed compared to the blank control, but marginally between the substrates. Most group differences compared to the blank control were detected after the fermentation of wheat bran, followed by the DFR_ms_, DFR_wms_ and beet pulp, indicating the highest fermentative capacity for these substrates. As the effects of the bacterial fermentation were more pronounced for the raw than for the enzymatically pre-digested food residues, it can be assumed that the raw food residues contained notable amounts of digestible nutrients, which were also microbially fermented when the raw substrates were inoculated, but which were available to a lesser extent in the pre-digested substrates. This might concern protein, as already discussed above, but also digestible carbohydrates, especially starch.

Interestingly, high concentrations of n-butyrate were measured after the inoculation of both raw and enzymatically pre-digested food residues. Butyrate is the major energy source for colonocytes [38] and also associated with beneficial effects on gut and host health [39]. Thus, the observed increase of n-butyrate when the food residues were microbially fermented can be considered as a positive result. When compared with the bacterial fermentation of the other test substrates, only enzymatically pre-digested wheat bran also increased the n-butyrate concentrations in the inoculum compared to the blank control. This observation is in contrast with results from Bosch et al. [6], where the incubation of beet pulp with canine feces for 72 hours resulted in higher butyrate concentrations than the incubation of wheat fiber. However, Tuncil et al. [40] also measured high butyrate concentrations, when wheat bran was incubated with human feces for 24 and 48 hours. In addition, the authors could demonstrate that the particle size of wheat bran affected its fermentative capacity [40]. Thus, the observed differences between the results of the present study and the study of Bosch et al. [6] might be attributed to differences in the study design or the test substrates used.

In the present study, the test substrates were incubated for 24 hours, which is in accordance with the protocol of Vierbaum et al. [13]. However, the incubation time in comparable studies varied from 3–72 hours [6, 27–30, 33], making data comparison difficult. In addition, as beet pulp, carrot pomace and brewer´s spent grains are by-products of the food industry, their composition might differ depending on the production processes. Although Serena and Bach Knudsen [41] could demonstrate that those by-products showed only moderate variations in the nutrient composition, even minor differences might influence the microbial fermentation of the substrates and should be taken into account when comparing different study results. With regard to food residues, it can be assumed that the composition might vary depending on the collection procedure. In the present study, two different batches of hotel catering leftovers were evaluated, which also differed in their heat treatment (sterilized vs. non-sterilized). For the potential future use of food residues for animal nutrition, a heat treatment might be necessary in order to improve the hygienic quality of the food residues and therefore to prevent health risks for the animals. In the present study, the sterilization process did not affect the fermentation of the raw food residues. In addition, although the composition differed between the two batches, comparable effects for the microbial fermentation of the raw food residues could be detected. For some variables, however, smaller effects were observed for the raw DFR_wms_. When the enzymatically pre-digested food residues were microbially fermented, the effects were more pronounced for the DFR_ms_ and DFR_wms_ than for the DFR_m_. It can be speculated that the heat treatment of the food residues might have affected the nutrient availability, but given the small sample size, this hypothesis should be further investigated in future studies. Both regarding the impact on the intestinal microbiota and the calculation of well-defined diets, compositional variability of food residues should be reduced if considered as a potential “new” ingredient for pet food in the future. In particular, collection and heat treatment procedures should be standardized.

For the interpretation of the results, a potential impact of the donor animals should finally be considered. The composition of the intestinal microbiota of dogs is dependent on animal related (breed, age), but also external (housing, diet) factors [42]. In this context, it has been demonstrated that differences in the *in vitro* fermentation of fiber substrates occurred, when the donor animals were either adapted to a diet with fermentable or non-fermentable fiber [43]. In the present study, feces of dogs kept under the same housing and feeding conditions were used for the *in vitro* experiments. The results, however, require a careful interpretation, taking into account that varying factors might affect the fermentative activity of the intestinal microbiota.

## Conclusions

Based on the present *in vitro* fermentation of raw and enzymatically pre-digested food residues, it can be assumed that food residues might contain both enzymatically digestible and microbially fermentable nutrients. In comparison with the other test substrates, the microbial fermentation of food residues was comparable or partially more pronounced, but differences between the two batches of food residues were also observed. A standardization of the collection and processing of food residues might be necessary if considered as a potential “new” ingredient for pet food in the future.

## Supporting information

S1 Data (XLSX)
